# The challenges of modeling and forecasting the spread of COVID-19

**DOI:** 10.1073/pnas.2006520117

**Published:** 2020-07-02

**Authors:** Andrea L. Bertozzi, Elisa Franco, George Mohler, Martin B. Short, Daniel Sledge

**Affiliations:** ^a^Department of Mathematics, University of California, Los Angeles, CA 90095;; ^b^Department of Mechanical and Aerospace Engineering, University of California, Los Angeles, CA 90095;; ^c^Department of Bioengineering, University of California, Los Angeles, CA 90095;; ^d^Department of Computer Science, Indiana University–Purdue University Indianapolis, Indianapolis, IN 46202;; ^e^Department of Mathematics, Georgia Institute of Technology, Atlanta, GA 30332;; ^f^Department of Political Science, University of Texas at Arlington, Arlington, TX 76019

**Keywords:** COVID-19, pandemic, branching process, compartmental models

## Abstract

The coronavirus disease 2019 (COVID-19) pandemic has placed epidemic modeling at the forefront of worldwide public policy making. Nonetheless, modeling and forecasting the spread of COVID-19 remains a challenge. Here, we detail three regional-scale models for forecasting and assessing the course of the pandemic. This work demonstrates the utility of parsimonious models for early-time data and provides an accessible framework for generating policy-relevant insights into its course. We show how these models can be connected to each other and to time series data for a particular region. Capable of measuring and forecasting the impacts of social distancing, these models highlight the dangers of relaxing nonpharmaceutical public health interventions in the absence of a vaccine or antiviral therapies.

## Significance

The coronavirus disease 2019 (COVID-19) pandemic has placed epidemic modeling at the forefront of worldwide public policy making. Nonetheless, modeling and forecasting the spread of COVID-19 remain a challenge. Here, we present and detail three regional-scale models for forecasting and assessing the course of the pandemic. This work is intended to demonstrate the utility of parsimonious models for understanding the pandemic and to provide an accessible framework for generating policy-relevant insights into its course. We show how these models can be connected to each other and to time series data for a particular region. Capable of measuring and forecasting the impacts of social distancing, these models highlight the dangers of relaxing nonpharmaceutical public health interventions in the absence of a vaccine or antiviral therapies.

The world is in the midst of an ongoing pandemic, caused by the emergence of a novel coronavirus. Pharmaceutical interventions such as vaccination and antiviral drugs are not currently available. Over the next year, addressing the coronavirus disease 2019 (COVID-19) outbreak will depend critically on the successful implementation of public health measures including social distancing, shelter in place orders, disease surveillance, contact tracing, isolation, and quarantine (1, 2). On 16 March, Imperial College London released a report (3) predicting dire consequences if the United States and the United Kingdom did not swiftly take action against the pandemic. In both nations, governments responded by implementing more stringent social distancing regulations (4). We now have substantially more case data from the United States, as well as the benefit of analyses performed by scientists and researchers across the world (5–12). Nonetheless, modeling and forecasting the spread of COVID-19 remain a challenge.

Here, we present three basic models of disease transmission that can be fit to data emerging from local and national governments. While the Imperial College study employed an agent-based method (one that simulates individuals getting sick and recovering through contacts with other individuals in the population), we present three macroscopic models: 1) exponential growth, 2) self-exciting branching process, and 3) the susceptible–infected–resistant (SIR) compartment model. These models have been chosen for their simplicity, minimal number of parameters, and for their ability to describe regional-scale aspects of the pandemic. In presenting these models, we demonstrate how they are connected and note that in different cases one model may fit better than another. Because these models are parsimonious, they are particularly well suited to isolating key features of the pandemic and to developing policy-relevant insights. We order them according to their usefulness at different stages of the pandemic—exponential growth for the initial stage, a self-exciting branching process when one is still analyzing individual count data going into the development of the pandemic, and a macroscopic mean-field model going into the peak of the disease. The branching process can also track changes over time in the dynamic reproductive number.

These models highlight the significance of fully implemented and sustained social distancing measures. Put in place at an early stage, distancing measures that reduce the virus’s reproduction number—the expected number of individuals who an infected person will spread the disease to—may allow much-needed time for the development of pharmaceutical interventions. By slowing the speed of transmission, such measures may also reduce the strain on health care systems and allow for higher-quality treatment for those who become infected. Importantly, the economic consequences of such measures may lead political leaders to consider relaxing them. The models presented here, however, demonstrate that relaxing these measures in the absence of pharmaceutical interventions may allow the pandemic to reemerge. Where this takes place, social distancing efforts that appear to have succeeded in the short term will have little impact on the total number of infections expected over the course of the pandemic.

The epidemiological perspective on modeling infectious disease spread involves consideration of a larger number of modeling parameters detailing the spread of and recovery from the disease, additional compartments corresponding to age categories, and other related choices (e.g., refs. 3 and 13). A data-driven approach to modeling COVID-19 has also emerged, in which statistical and machine learning models are used for forecasting cases, hospitalizations, deaths, and impacts of social distancing (14, 15). Our work demonstrates the utility of parsimonious epidemic models for understanding the pandemic and provides an accessible framework for a larger group of quantitative scientists to follow and forecast the COVID-19 pandemic. It includes explanations that will help allow scientific researchers to develop insights that may contribute to public health policy making, including contributing to public health forecasting teams. Importantly, the branching process model that we detail is relatively new and underutilized in epidemiology. It provides a method for quantitatively estimating dynamic reproduction numbers, which can be critical in assessing the impact of distancing measures over time. The results from the parsimonious models presented here are consistent with recent analyses from public health officials in California (16) and with the original Imperial College model (3).

We present examples of forecasts for viral transmission in the United States. Where other studies have typically developed and presented one model (choosing to fit parameters within the chosen model), our analysis compares three different forecasting models using a fitting criterion. The results of these models differ depending on whether the data employed cover confirmed cases or mortality. In addition, many aspects of disease spread, such as incubation periods, fraction of asymptomatic but contagious individuals, seasonal effects, and the time between severe illness and death, are not considered here. In some cases (e.g., seasonal), relevant data do not exist, and in other cases (e.g., age of patients), we choose not to include additional parameters in favor of parsimony.

## Results

### A. Exponential Growth.

**Fig. 1.**
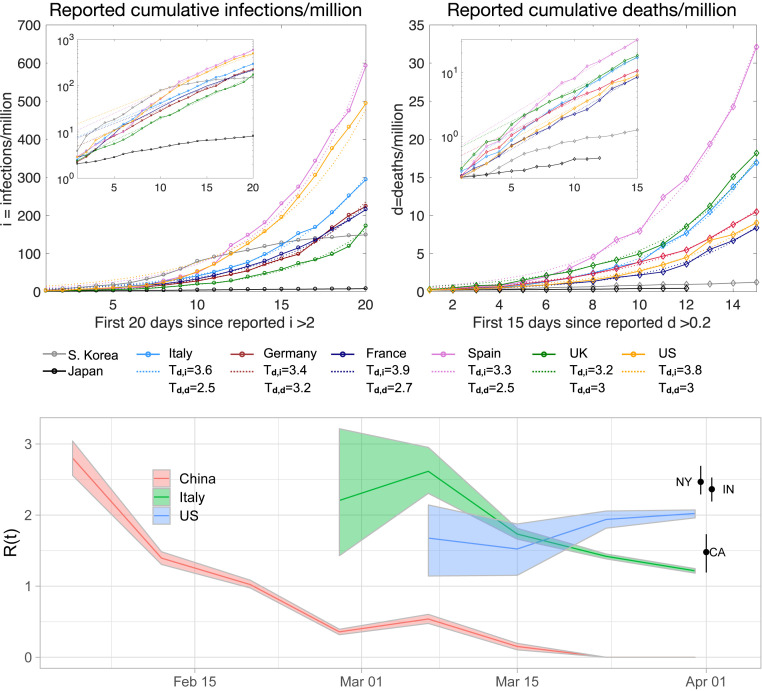
(*Upper*) Exponential model applied to new infection and death data for Italy, Germany, France, Spain, the United Kingdom, and the United States, normalized by the total country population (54). *Insets* show the same data on a logarithmic scale. Both the normalized infection i and death d data were thresholded to comparable initial conditions for each country; fits are to the first 15 to 20 d of the epidemic after exceeding the threshold. The fitted doubling time is shown for both infections ($T_{d,i}$) and death ($T_{d,d}$) data. Data from Japan and South Korea are shown for comparison. (*Lower*) Dynamic reproduction number (mean and 95% CI) of COVID-19 for China, Italy, and the United States estimated from reported deaths (17) using a nonparametric branching process (18). Current estimates are as of 1 April 2020 of the reproduction number in New York (NY), California (CA), and Indiana (IN; confirmed cases used instead of mortality for Indiana). Reproduction numbers of COVID-19 vary in different studies and regions of the world (in addition to over time) but have generally been found to be between 1.5 and 6 (19) prior to social distancing.

Epidemics naturally exhibit exponential behavior in the early stages of an outbreak, when the number of infections is already substantial but recoveries and deaths are still negligible. If at a given time t there are I(t) infected individuals and α is the rate constant at which they infect others, then at early times (neglecting recovered individuals), $I\left(t\right)=I_{0}e^{\alpha t}$. The time it takes to double the number of cumulative infections (doubling time) is a common measure of how fast the contagion spreads: if we start with Ī infections, then at time $T_{d}=\ln2/\alpha$ we achieve 2Ī infections. For the COVID-19 outbreak, exponential growth is seen in data from multiple countries (Fig. 1), with remarkably similar doubling times in the early stages of the epidemic. For COVID-19, we expect an exponential growth phase during the first 15 to 20 d of the outbreak in the absence of public health interventions such as social distancing, isolation, or quarantine. This estimate is based on patient data from the Wuhan outbreak, which indicate that the average time from illness onset to death or discharge is between 17 and 21 d for hospitalized patients (20, 21). Because deaths are a fraction of infections, they initially increase at a similar exponential pace, with some delay relative to the beginning of the outbreak. These observed doubling time estimates (2 to 4 d) are significantly smaller than early estimates (∼7 d) obtained using data collected in Wuhan (22).

### B. Self-Exciting Branching Process.

A branching point process (23–25) can also model the rate of infections over time. Point process models are data driven and allow for parametric or nonparametric estimation of the reproduction number and transmission timescale. They also facilitate estimation of the probability of extinction at early stages of an epidemic. These models have been used for various social interactions including spread of Ebola (26), retaliatory gang crimes (27), and email traffic (28, 29). The intensity (rate) of infections can be modeled as

$$\lambda \left(t\right)=\mu +\underset{t_{i}<t}{\sum }R\left(t_{i}\right)w\left(t-t_{i}\right),$$ [1]

where t is the current time and $t_{i}$ are the times of previous infection incidents. Here, the dimensionless reproduction number, R(t), evolves in time (18, 30–33) to reflect changes in disease reproduction in response to public health interventions (e.g., school closings, social distancing, closures of nonessential businesses, isolation, and quarantine). The distribution of interevent times $w\left(t_{i}-t_{j}\right)$ is a gamma or Weibull distribution (11, 33, 34) with shape parameter k and scale parameter b. Finally, the parameter μ allows for exogenous infection cases. The point process in Eq. **1** is an approximation to the common SIR model of infectious diseases (described later) during the initial phase of an epidemic when the total number of infections is small compared with the overall population size (35).

Given Eq. **1**, the quantity

$$p_{ij}=R\left(t_{j}\right)w\left(t_{i}-t_{j}\right)/\lambda \left(t_{i}\right)$$ [2]

gives the probability of secondary infection i having been caused by primary infection j. The dynamic reproduction number R(t) can then be estimated via expectation–maximization (18) using a histogram estimator:

$$R\left(t\right)=\overset{B}{\underset{k=1}{\sum }}r_{k}1\left\{t\in I_{k}\right\}.$$ [3]

Here, $I_{k}$ are intervals discretizing time, and B is the number of such intervals. The reproduction number, $r_{k}$, in each interval k is determined by

$$r_{k}=\underset{t_{i}>t_{j}}{\sum }p_{ij}1\left\{t_{j}\in I_{k}\right\}/N_{k},$$ [4]

where $N_{k}$ is the total number of events in interval k.

Fig. 1, *Lower* shows the estimated dynamic reproduction number (36, 37) of COVID-19 in China, Italy, and the United States during the early stage of the pandemic, from late January 2020 to early April 2020. The branching point process is fit to mortality data (17) using an expectation–maximization algorithm (18). Public health measures undertaken in China appear to have reduced R(t) to below the self-sustaining level of R=1 by the middle of February. In Italy, public health measures brought the local value of R(t) down; as of early April, however, it remained above R=1. The estimated reproduction number in the United States as a whole stood at around 2.5. The reproduction number, however, varies notably by location.

This model can be adapted to capture the long-term evolution of the pandemic by incorporating a prefactor that accounts for the dynamic decrease in the number of susceptible individuals (35):

$$\lambda^{h}\left(t\right)=\left(1-I_{c}\left(t\right)/N\right)\left(\mu +\underset{t_{i}<t}{\sum }R\left(t_{i}\right)w\left(t-t_{i}\right)\right).$$ [5]

Here, $I_{c}\left(t\right)$ is the cumulative number of infections that have occurred up to time t, and N is the total population size. This version of the branching process model, referred to as HawkesN, represents a stochastic version of the SIR model; with large R, the results of HawkesN are essentially deterministic. When projecting, we use our estimated $R\left(t_{i}\right)$ at the last known point for all times going forward. Since the $N_{t}$ term is the number of infections, if our estimates for $R\left(t_{i}\right)$ are based on mortality numbers, we must also choose a mortality rate to interpolate between the two counts; although existing estimated rates vary significantly, we choose 1% as a plausible baseline (38) (discussion in *Materials and Methods*). Alternatively, we also create forecasts for three US states based on fits to reported case data (Table 1).

**Table 1.** Model Fits to Early Time Data

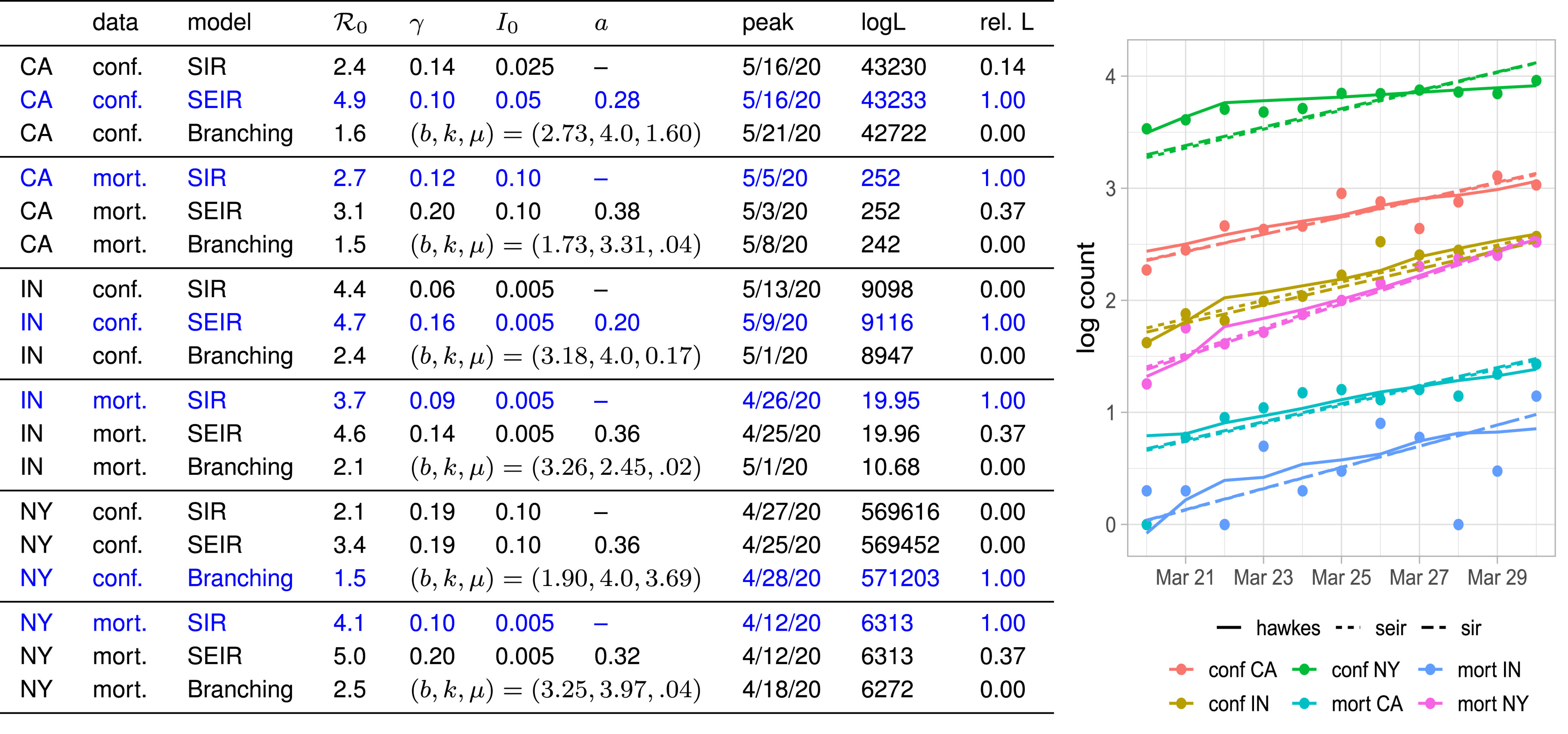

(*Left*) Fit of confirmed case (conf.) or mortality (mort.) data from California (CA), Indiana (IN), and New York (NY) states to three different models (SIR, SEIR, and branching process) using Poisson regression. The table shows the log likelihood and the relative likelihood exp($\left(AIC_{min}-AIC\right)=2$) based on the Akaike information criteria (AIC) (39). The blue lettering corresponds to the lowest AIC value. The branching process parameters include a Weibull shape k and scale b for w(t), along with the exogenous rate μ. The table also shows parameters from the fit and the projected date for the peak in new cases for each of these datasets; the projected peak date for the branching process is made using the HawkesN model. For each state, we run the fit on both confirmed case data and mortality data, taken from ref. 17. (*Right*) Shown are the actual data points compared with the fitted curves.

### C. Compartmental Models.

The SIR model (40–42) describes a classic “compartmental” model with SIR population groups. A related model, susceptible–exposed–infected–resistant (SEIR), includes an “exposed” compartment that models a delay between exposure and infectiousness. The SEIR model was shown to fit historical death record data from the 1918 influenza epidemic (43), during which governments implemented extensive social distancing measures, including bans on public events, school closures, and quarantine and isolation measures. The SIR model can be fit to the predictions made in ref. 3 for agent-based simulations of the United States. The SIR model assumes a population of size N where S is the total number of susceptible individuals, I is the number of infected individuals, and R is the number of resistant individuals. For simplicity of modeling, we view deaths as a subset of resistant individuals, and deaths can be estimated from the dynamics of R; this is reasonable for a disease with a relatively small death rate. We also assume a timescale short enough such that humans’ natural resistance to the disease does not introduce new susceptible people after recovery.

The SIR model equations are

$$\frac{dS}{dt}=-\beta \frac{IS}{N},\;\frac{dI}{dt}=\beta \frac{IS}{N}-\gamma I,\;\frac{dR}{dt}=\gamma I.$$ [6]

Here, β is the transmission rate constant, γ is the recovery rate constant, and $R_{0}=\beta /\gamma$ is the reproduction number. One integrates Eq. **6** forward in time from initial values of S, I, and R at time 0. The SEIR model includes an exposed category E:

$$\begin{array}{ll} \frac{dS}{dt} & =-\beta \frac{IS}{N},\;\frac{dE}{dt}=\beta \frac{IS}{N}-aE,\\ \frac{dI}{dt} & =aE-\gamma I,\;\frac{dR}{dt}=\gamma I. \end{array}$$

Here, a is the inverse of the average incubation time. Both models are fit, using maximum likelihood estimation with a Poisson likelihood, to data for three US states (California, New York, and Indiana) (17). Table 1 compares the results with the branching process. We use the relative likelihood based upon the Akaike Information Criterion (AIC) (39) to measure model performance for each dataset; AIC is biased against models with more parameters. The SEIR model performs better on the confirmed data for California and Indiana, possibly due to the larger amount of data, compared with mortality for which SIR is the best for all three states. The branching process performs best for confirmed cases in New York. Our choice of fitting follows the method in ref. 43 for the 1918 pandemic death data. Our focus is on model comparison rather than measuring uncertainty of parameters in a specific model, as is currently being done for hospital demand forecasts in Los Angeles (16). For interval forecasts with uncertainty quantification, one may consider a negative binomial alternative to Poisson regression that captures overdispersion in case and death counts (44, 45).

**Fig. 2.**
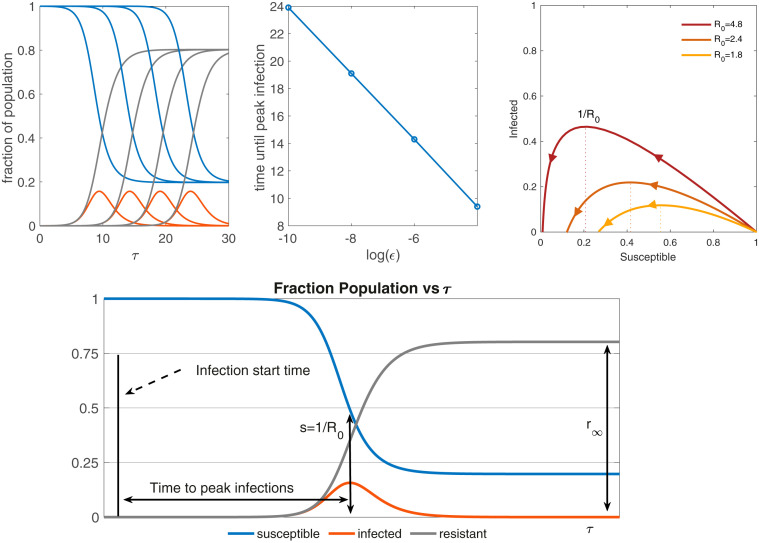
Solution of the dimensionless SIR model (5) with $R_{0}=2$. *Upper Left* shows the graphs of s (blue), i (orange), and r (gray) on the vertical axis vs. τ on the horizontal axis, for different ϵ. The corresponding values of ϵ from left to right are $10^{-4}$, $10^{-6}$, $10^{-8}$, $10^{-10}$, respectively. *Upper Center* shows the time until peak infections vs. log(ϵ) for the values shown in *Upper Left*. This asymptotic tail to the left makes it challenging to fit data to SIR in the early stages. *Upper Right* is a phase diagram for fraction of infected vs. fraction of susceptible with the direction of increasing τ indicated by arrows, for three different values of $R_{0}$. *Lower* displays a typical set of SIR solution curves over the course of an epidemic, with important quantities labeled.

**Fig. 3.**
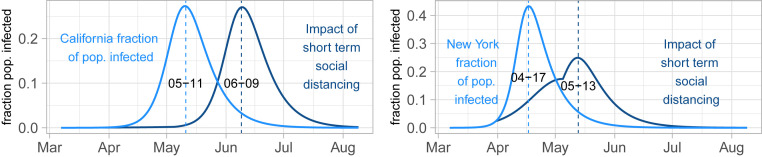
Scenarios for the impact of short-term social distancing: fraction of population vs. date. (*Left*) California SIR model based on mortality data with parameters from Table 1 ($R_{0}=2.7$, γ=.12, $I_{0}=.1$) under two scenarios: $R_{0}$ constant in time (light blue) and $R_{0}$ cut in half from 27 March (1 wk from the start of the California shutdown) to 5 May but then returned to its original value, to represent a short-term distancing strategy (dark blue). (*Right*) New York SIR model with parameters from Table 1 ($R_{0}=4.1$, γ=.1, $I_{0}=05$) under the same two scenarios but with short-term distancing occurring over the dates of 30 March (1 wk from the start of the New York shutdown) to 5 May. In both states, the distancing measures suppress the curve and push the peak infected date into the future, but the total number of cases is only slightly reduced.

We can further understand the role of parameters in our models via a dimensionless formulation of Eq. **6**. There are two timescales dictated by β and γ. Therefore, if time is rescaled by γ to τ=γt and s=S/N, i=I/N, and r=R/N represent fractions of the population in each compartment, then in the case of a novel outbreak with no initially resistant individuals, we obtain

$$\begin{array}{ll} \frac{ds}{d\tau }=-R_{0}is,\;\frac{di}{d\tau }=R_{0}is-i,\;\frac{dr}{d\tau } & =i,\\ \left(s,i,r\right){|}_{\tau =0} & =\left(1-\epsilon ,\epsilon ,0\right), \end{array}$$ [7]

where 0<ϵ<<1 is the initial fraction of the infected population at the start time, and the system retains only one dimensionless parameter $R_{0}$ that, in conjunction with the initial conditions, completely determines the resulting behavior. For Eq. **7**, the shapes of the solution curves s(τ),i(τ),r(τ) do not depend on ϵ, other than exhibiting a time shift that depends logarithmically on ϵ (Fig. 2). This is a universal solution for the SIR model in the limit of small ϵ (Fig. 3), depending only on $R_{0}$. Critically, the height of the peak in i(t) and the total number of resistant/susceptible people by the end of the epidemic are determined by $R_{0}$ alone. However, the sensitivity of the time translation to the parameter ϵ and the dependence of true time values of the peak on parameter γ make SIR challenging to fit to data at the early stages of an epidemic when Poisson statistics and missing information are prevalent. When using early-time death data to fit SIR, the estimate of the percentage of deaths per total number of infections (chosen as 1% here) has a sensitivity that can be understood directly in terms of this shift in the time to peak. This is important information for public health officials, policy makers, and for political leaders interested in decreasing $R_{0}$ for substantial periods of time. This sensitivity to parameters helps explain why projections of the outbreak can display such large variability and highlights the need for extensive disease testing within the population to more accurately track the epidemic curve. After the surge in infections, the model asymptotes to an end state in which r approaches the end value $r_{\infty }$ and s approaches $1-r_{\infty }$ and the infected population approaches 0. The value $r_{\infty }$ satisfies a well-known transcendental equation (46–48). A phase diagram of the universal solutions for several $R_{0}$ values is shown in Fig. 2, *Upper Right*. The dynamics start in the bottom right corner where s is almost 1 and follow the colored line to terminate on the i=0 axis at the value $s_{\infty }$. A rigorous derivation of the limiting state under the assumptions here can be found in refs. 46–48.

Under the SIR (and similar) model(s), if $R_{0}$ is decreased during the middle of an outbreak, through social distancing and other public health measures, the rate of new infections will decrease. However, unless the number of infected individuals is brought down to zero, the outbreak will likely reemerge, and the total number of infections may still be a large fraction of the population. This is illustrated in Fig. 3, where we present scenarios of no social distancing vs. short-term social distancing with parameters from Table 1 for death data up through the end of March, fit to the SIR model. We caution that the goal of these scenarios is not to produce highly accurate percentages but rather, to present scenarios under different basic assumptions that illustrate the usefulness of social distancing measures and the potential danger in easing them too soon.

For the New York state scenario, with $R_{0}$ presumed to decrease by a factor of two with distancing measures, the outbreak is not completely controlled by distancing, and the number of infections continues to rise to approximately 10% by mid-April. In contrast, without distancing measures the scenario shows four times that number of infected individuals by mid-April, a level that would have represented a potentially disastrous strain on the hospital system. In the California scenario, distancing measures bring the effective $R_{0}$ closer to one, thus keeping infections at a much smaller portion of the population than the scenario with no social distancing, at least during the period when the distancing is still in effect. We take these scenarios one step further by suddenly stopping distancing on 5 May (this is both extreme and hypothetical, but it serves to illustrate the model). Because of the low initial infected count in California, bringing $R_{0}$ back to the original predistancing level produces a curve that follows the original peak scenario, just shifted later in time. For New York state, because a nontrivial fraction of the population is initially infected, there are fewer to infect, and the new peak is less steep than the scenario without any distancing.

## Discussion

Our analysis, employing parsimonious models, illustrates several key points. 1) The reproduction number R is highly variable both over time and by location, and this variability is compounded by distancing measures. These variations can be calculated using a stochastic model, and lower R is critical to decreasing strains on health care systems and to creating time to develop effective vaccines and antiviral therapies. 2) Mortality data and confirmed case data have statistics that vary by location and by time depending on testing and on accurate accounting of deaths due to the disease. Differences in collection methods and in the accuracy of morbidity and mortality data can lead to different projected outcomes. 3) Nonpharmaceutical public health interventions (NPIs) such as social distancing and shelter in place orders offer an important means of reducing the virus’s reproduction number. Nonetheless, NPIs may not have a substantial impact on the total number of infections unless sustained over time. Policy makers should be cautious about scaling back distancing measures after early signs of effectiveness.

During the 1918 influenza pandemic, the early relaxation of social distancing measures led to a swift uptick in deaths in some US cities (43). The models presented here help to explain why this effect might occur, as illustrated in Fig. 3. Already, policy makers in many jurisdictions have started to implement new social protocols that allow for increased economic activity. In the United States, where public health authority is vested largely in states and localities, key decisions about such measures will be in the hands of local officials, with national agencies such as the Centers for Disease Control and Prevention playing a coordinating role and offering guidance (49). Nationally, policy makers may also consider funding mechanisms and regulatory measures that might facilitate a more unified approach to the pandemic (50), as well as fiscal measures aimed at ameliorating the economic effects of social distancing and shelter in place orders.

Of the models presented here, only the point process model tracks the reproductive number changing over time (although compartmental models can be modified to do the same). Dynamic change in the reproductive number is the major challenge in forecasting such a pandemic. Some new approaches to forecasting long-term trends in the COVID-19 pandemic attempt to address this concern by fitting curves in countries in the later stages of spread (after social distancing) and then applying those fitted models to regions in earlier stages (15). The long-term SIR curves in Fig. 3 are idealized counterfactuals in the event of no social distancing, rather than forecasts of the actual peak date and total infected. Moreover, in the absence of distancing policies, people may still choose to distance out of fear driven by an upswing in recent deaths, as was modeled in ref. 43 in the 1918 pandemic.

The models presented here are parsimonious, making a variety of assumptions in order to increase understanding and to avoid overfitting the limited and incomplete data available; more complex models have been introduced and are currently in use (3, 13). Even with these simple models, however, the parameters obtained from our fits (Table 1) can vary significantly for a given location. Although we have in each case determined which of these fits appears to have most validity, in many cases these are not strong indicators. These models have several sources of uncertainty, including parameter uncertainty, variation based on data or model type used, and most importantly, uncertainty in the severity and length of social distancing measures, which can change the peak date by months or even create multiple peaks. This variability in outcomes highlights the challenges of modeling and forecasting the course of a pandemic during its early stages and with only limited data. This uncertainty is a major challenge for policy makers, who must consider the social and economic consequences of disruptive public health interventions while recognizing that relaxing them may swiftly lead to the reemergence of a devastating disease.

## Materials and Methods

### Relation between the Exponential Model and Compartment Models.

The exponential model is appropriate during the first stages of the outbreak, when recoveries and deaths are negligible: in this case, the SIR compartment model can be directly reduced to an exponential model. If we assume S≈N in Eq. **6**, then dI(t)/dt≈(β−γ)I, with the exponential solution $I\left(t\right)=I_{0}e^{\alpha t}$ with α=β−γ and $I_{0}$ the initial number of infections. We expect at very early times t≪1/γ that the recovery will lag infections so one might see α∼β at very early times and then reduce to α∼β−γ after t>1/γ. Reports and graphs disseminated by the media typically report cumulative infections, which include recoveries and deaths. Using the SIR model, the total cumulative infections are $I_{c}\left(t\right)=I\left(t\right)+R\left(t\right)$ and evolve as $dI_{c}\left(t\right)/dt=\beta sI$. Integrating this, we see that $I_{c}$ likewise grows exponentially with the same rate α=β−γ. An important observation is that the doubling time for cumulative infections [$T_{d}=\ln\left(2\right)/\alpha$] will change during the early times, with a shorter doubling time when t≪1/γ and a longer doubling time when t>1/γ.

### Relation between the HawkesN and SIR Models.

Following refs. 35 and 51, first a stochastic SIR model can be defined where a counting process $I_{c}\left(t\right)=N-S\left(t\right)$ tracks the total number of infections up to time t, N is the population size, and S(t) is the number of susceptible individuals. The process satisfies

P(dC(t)=1)=βS(t)I(t)dt/N+o(dt)

P(dR(t)=1)=γI(t)dt+o(dt),

which then gives the rate of new infections and new recoveries as (35)

$$\lambda^{I}\left(t\right)=\beta S\left(t\right)I\left(t\right)/N,\;\lambda^{R}\left(t\right)=\gamma I\left(t\right).$$

It is shown in ref. 51 that the continuum limit of the counting process approaches the solution to the SIR model in Eq. **6**. Furthermore, for an exponential kernel w(t) in the HawkesN model in Eq. **5** with parameter γ and constant reproduction number ($R_{0}$), then $E\left[\lambda^{I}\left(t\right)\right]=\lambda^{H}\left(t\right)$ where μ=0, $\beta =R_{0}\gamma$ (ref. 35 has further details).

### Fitting the SIR, SEIR, and Branching Process Models.

The parameters for SIR and SEIR in Table 1 were found using maximum Poisson likelihood regression (as in ref. 43 for death data from the 1918 pandemic in US cities) via grid search with ranges $I_{0}\in \left[.005,.1\right]$, $R_{0}\in \left[1.5,5\right]$, γ∈[.01,.2], and μ∈[.01,.4].

The branching process was fit using a nonparametric expectation maximization algorithm, the details of which can be found in ref. 18. Models were fit to empirical new infections per day or new mortality counts per day. We assumed that 1% of those labeled as resistant in the simulations were fatal cases. For morality rates in the range of 0.3 to 3%, the peak date changes by up to 2 wk. Data quality is an issue during the COVID-19 pandemic due to lack of uniform testing. Uncertainty in estimates of the total number infected leads to uncertainty in forecasting results. The likelihood is somewhat flat at the maximum for models in Table 1, with multiple parameter combinations yielding plausible fits. Fitting SIR type models is known to be challenging due to parameter identifiability issues (52, 53). However, the peak date only varies by 1 to 3 d for parameters within two units of the maximum log likelihood.

## Data Sources

The data used in this manuscript were downloaded on 1 April 2020 from ref. 54 for Fig. 1, *Upper*, 16 June 2020 from ref. 17 for Fig. 1, *Lower*, and on 2 April 2020 from ref. 17 for Table 1. Data from both refs. 17 and 54 are publicly available. Case and death counts for COVID-19, such as those reported in ref. 17 that are used in the present study, are well known to suffer from ascertainment issues (55). The data also have other sources of error due to the nature in which they are aggregated from primary sources, where reporting is often lagged. Policy decisions based upon models fit to these data must take these ascertainment and data quality issues into account. The code and data used to generate Fig. 1, *Upper* can be downloaded from GitHub at https://github.com/francoelisa/PNAS2020. The code to download data and generate Fig. 1, *Lower* can be downloaded from GitHub at https://github.com/gomohler/pnas2020/tree/master/dynamic_R. The code to generate Table 1 is available in GitHub at https://github.com/gomohler/pnas2020.
